# Wavelet radiomics features from multiphase CT images for screening hepatocellular carcinoma: analysis and comparison

**DOI:** 10.1038/s41598-023-46695-8

**Published:** 2023-11-10

**Authors:** Van Ha Tang, Soan T. M. Duong, Chanh D. Tr. Nguyen, Thanh M. Huynh, Vo T. Duc, Chien Phan, Huyen Le, Trung Bui, Steven Q. H. Truong

**Affiliations:** 1 VinBrain JSC., 458 Minh Khai, Hanoi, 11619 Vietnam; 2 Le Quy Don Technical University, 236 Hoang Quoc Viet, Hanoi, 11917 Vietnam; 3 VinUniversity, Vinhomes Ocean Park, Hanoi, 12406 Vietnam; 4 University Medical Center Ho Chi Minh City, 215 Hong Bang, Ho Chi Minh City, 12406 Vietnam; 5 Adobe Research, San Francisco, CA 94103 USA

**Keywords:** Cancer screening, Classification and taxonomy, Computational models, Machine learning

## Abstract

Early detection of liver malignancy based on medical image analysis plays a crucial role in patient prognosis and personalized treatment. This task, however, is challenging due to several factors, including medical data scarcity and limited training samples. This paper presents a study of three important aspects of radiomics feature from multiphase computed tomography (CT) for classifying hepatocellular carcinoma (HCC) and other focal liver lesions: wavelet-transformed feature extraction, relevant feature selection, and radiomics features-based classification under the inadequate training samples. Our analysis shows that combining radiomics features extracted from the wavelet and original CT domains enhance the classification performance significantly, compared with using those extracted from the wavelet or original domain only. To facilitate the multi-domain and multiphase radiomics feature combination, we introduce a logistic sparsity-based model for feature selection with Bayesian optimization and find that the proposed model yields more discriminative and relevant features than several existing methods, including filter-based, wrapper-based, or other model-based techniques. In addition, we present analysis and performance comparison with several recent deep convolutional neural network (CNN)-based feature models proposed for hepatic lesion diagnosis. The results show that under the inadequate data scenario, the proposed wavelet radiomics feature model produces comparable, if not higher, performance metrics than the CNN-based feature models in terms of area under the curve.

## Introduction

According to Globocan 2020, liver malignancy is the sixth most common cancer overall and the third most prevalent cause of cancerous death in both genders^1^. Among primary liver cancers, the most frequently encountered is hepatocellular carcinoma (HCC)^2,3^, for which treatment plans are distinguished from the remaining entities. The most crucial factors in enhancing patient prognosis are early detection and accurate characterization of HCC^4,5^.

Over the past decade, there has been a growing interest in developing computer-aided diagnosis (CAD) of liver lesions based on medical image analysis. As the medical data of the hepatic lesions is typically scarce, the majority approaches for liver lesion diagnosis can be divided into two categories: deep convolutional neural networks (CNN) and radiomics features-based models. The deep CNN aims to enhance performances of liver lesions prediction and overcomes the issue of data scarcity by leveraging the knowledge learned from the similar image processing tasks. In particular, it uses deep CNN backbones well-trained on generic image datasets for the adaptation to the medical imaging domain, also known as a transfer learning (TL) technique. With application to HCC identification, several deep CNN models, including VGG^6^, ResNet-50^7^, GoogleNet^8^, and 3D ResNet-18^9^ have been employed and shown improved performances.

The radiomics features analysis of liver lesions, on the other hand, has captured numerous studies due to its capability of encoding informative biological information from medical images and handling the insufficient data scenarios^10–15^. Radiomics generally refers to the high-throughput extracting and selecting of quantitative image features, and subsequent mining them for clinical knowledge and application^16–18^. A typical radiomics features-based pipeline includes data acquisition, image segmentation, feature extraction and selection, as well as lesion classification. Data collection can be performed using CT, which is one of the most common and robust imaging techniques for the detection of liver cancer. Segmentation is performed either manually or automatically to yield the region of interest (ROI) of liver lesions used for subsequent steps. Extracting and selecting relevant and high-quality features play a crucial role in liver lesion classification and treatment^4,19,20^.

Several radiomics feature-based methods have been proposed for enhancing liver lesion classification performances^10,11,21–28^. These investigations mainly focus on feature extraction, feature selection, and liver lesion classification techniques. In^22–24^, shape and geometric features were extracted from original CT images for liver lesions differentiation. Similarly, histogram-based feature extraction followed by logistic regression (LR) and support vector machine (SVM) were used for the diagnosis of HCC and cirrhosis diseases in^11^. In^25–28^, first-order features characterizing the histogram of the ROI were used for early HCC detection. In addition, second-order texture descriptors of the gray level co-occurrence matrix (GLCM) were employed to characterize HCC in^13,28,29^.

Together with feature extraction, several feature selection and classification techniques were investigated for enhancing liver lesion identification. Among such feature selection models, principal component analysis (PCA) was prominently introduced to prune non-relevant features in^23,27,30^. Furthermore, filtering techniques, including variance-based and correlation-based statistical threshold, as well as the model-based approach using least absolute shrinkage and selection operator (LASSO) were investigated for improving feature representation and classification of HCC and hepatic hemangioma^20^. In terms of classifiers, techniques including SVM used in^13,22,30^, multilayer perceptron (MLP) neural network employed in^31,32^, and LR in^11,14^ were shown to be prominent in the present context for the binary classification of HCC and non-HCC (focal) lesions, called as non-HCC for short. It is worth noting here that although efforts have been made to exploit radiomics features for characterizing liver lesions, it is needed to investigate how to combine radiomics features extracted from different imaging modalities, enrich their representations in different imaging domains, and develop an efficient model for the feature selection and HCC and non-HCC classification.

In this paper, we consider three important issues in classifying HCC and non-HCC liver lesions: radiomics feature extraction, feature combination, and feature selection using the CT imaging model. We introduce the wavelet-transformed radiomics features and analyze their effects on the classification performance, especially when combined with those extracted from the original CT imaging domain. Our analysis shows significant performance improvement by combining relevant wavelet-domain texture features and important original-domain features. The combination and enrichment of radiomics features extracted from different domains and modalities enhance the classification capability, but also lead to a challenge for feature selection. To address this issue, we evaluate different approaches for feature selection, including filter-based, wrapper-based, and model-based methods, and propose a logistic sparsity-based regression model for efficient feature selection. Experimental results show that the proposed sparsity-based feature selection model is the most effective technique among the tested feature selection methods; it gives a more compact feature subset and yields significantly higher performance metrics for most prominent classifiers, including logistic sparsity-based regression, MLP, and SVM. To support this research, we have prepared a dataset that comprises CT data of 253 patients with manually segmented liver lesion ROI and their hepatic lesion labeling for HCC and non-HCC categorization. The key contributions of this paper are highlighted as follows:

1. We introduce wavelet-domain radiomics features derived from multiphase CT images to enrich the representations of different focal liver lesions (FLLs). We also analyze the effects of combining the wavelet and original CT image domain radiomics features on the HCC and non-HCC classification performance. Experimental results show that combining features extracted from the two domains significantly improves the discriminative capability compared to using only the wavelet-domain or original-domain features. Although wavelet radiomics features have been considered previously, this study, for the first time, introduces wavelet radiomics for the representations of FLLs imaging by multiphase contrast-enhanced CT modality and analyzes its effects on classifying HCC and non-HCC. Such analysis and comparison have not been investigated so far.
2. This paper proposes a new model for efficient radiomics feature selection. The introduced model employs a sparse representation to handle the ill-posed feature selection problem in which the number of studied samples is far fewer than the number of extracted radiomics features. Furthermore, the proposed model incorporates statistical logistic modeling to represent the target output’s conditional probability given the feature. We formulate the problem using the Bayesian framework and introduce an algorithm to solve the feature selection problem efficiently.
3. This study analyzes and compares the proposed logistic sparsity feature selection model with several other techniques, including filter-based, wrapper-based, model-based, and dimensional reduction methods. We find through the experiments that the proposed logistic sparsity model is capable of yielding a compact and relevant feature subset and outperforms the other feature selection approaches with statistical significance. Furthermore, under the limited number of training samples, the relevant wavelet radiomics features tend to generalize well and outperform several deep CNN-based feature models proposed for liver lesion diagnosis.
4. We have prepared and processed a CT dataset of 253 patients with hepatic lesions to support this study. The preparation comprises screening and annotating tasks. The former involves using both the clinical and pathological information first to select the patients of interest and then identify the types of liver lesions in each case chosen. The latter requires experienced radiologists to annotate the masks of hepatic lesions manually.

The remainder of the paper is organized as follows. In the “Materials” section, we present the CT dataset and its preparation and annotation for this study. This is followed by the “Methods” section, in which we introduce the proposed radiomics feature-based model for HCC and non-HCC discrimination, including feature extraction and selection, and classification algorithms. Next, the “Experimental results and discussion” section presents the experimental protocol, results, analysis, and discussion. Finally, in the “Conclusion” section, we give the concluding remarks.

**Figure 1:**
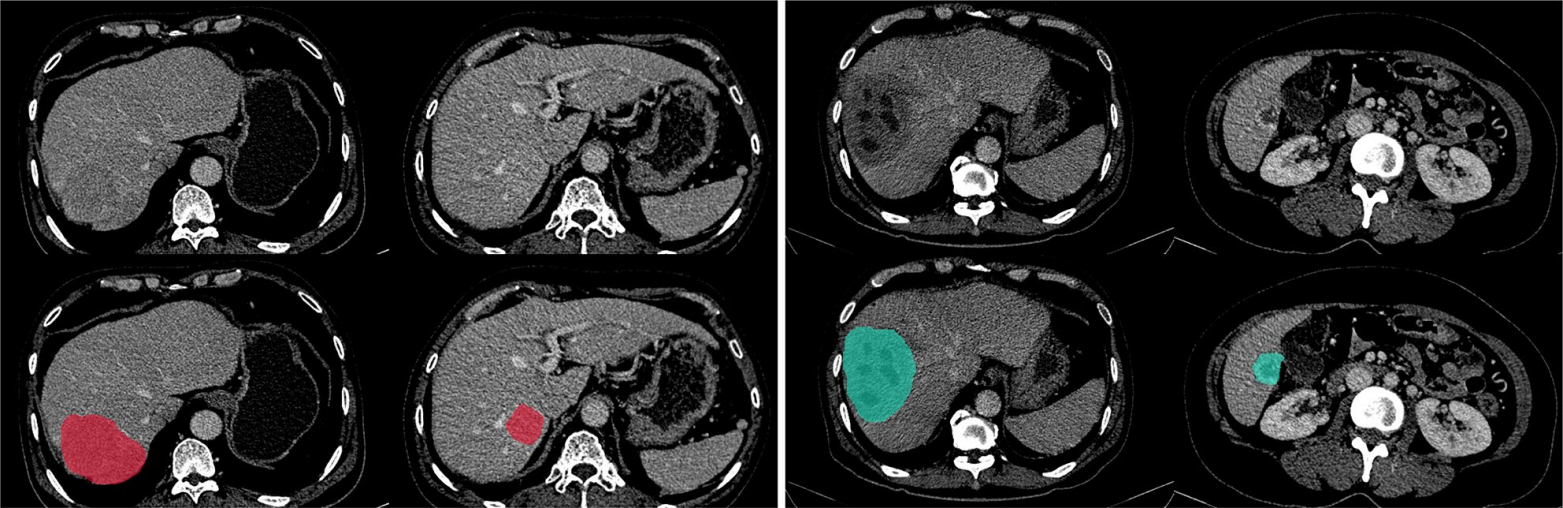
Examples of CT venous slices (top row) and the hepatic lesion annotation overlaid (bottom row) in out dataset. HCC tumors (red) are in the first two CT slices, and non-HCC lesions (cyan) are in the last two CT slices. Best view in color.

## Materials

This section presents the dataset used in this study. It first gives the patients and screening protocol and then presents data annotating and splitting.

### Participants and screening protocol

Using an institutional review board-approved protocol, we retrospectively collected data from patients with focal liver lesions, including cirrhosis, from March 2016 to November 2020 at the University Medical Center, Ho Chi Minh City. We randomly selected 380 patients from the study period. We then chose the data of patients with the correct multiphase abdominal CT and fewer than ten FLLs, resulting in a computed tomography dataset of 253 patients. The dataset has the age range from 18 to 86, and gender distribution (male|female) = 152|101.

All patients underwent the multiphase abdominal CT without any special preparation, e.g. bowel preparation or drinking oral contrast, using either 64-slice or 128-slice scanners (SOMATOM^®^ Definition AS/AS+, Siemens Healthineers AG, Erlangen, Germany). The multiphase scanning protocol, including non-contrast, arterial, venous, and delayed phases, was performed after the administration of intravenous contrast material (Xenetix^®^ 300 mgI/ml, Guerbet SA, Villepinte, France; Ultravist^®^ 300 mgI/ml, Bayer AG, Leverkusen, Germany; Omnipaque$^{\text{TM}}$ 300 mgI/ml, GE Healthcare Inc., Chicago, United States). The arterial phase was 30–35 s after the IV contrast injection, while the venous phase was 60–70 s and the delayed phase was 180 s. All 3D CT data were acquired with the slices in the axial plane, a size of 512 × 512, and pixel spacing ranging from 0.5 to 0.86 mm. The slice increment varied in the range (0.6–1.0) mm. The number of slices felt within a wide range, i.e. (175–830), as most patients underwent the abdomen CT scan while a few underwent the chest-abdomen CT scan for additional staging purposes.

### Hepatic lesion labeling and data splitting

The labeling process was conducted by radiologists with more than five years of experience in hepatic imaging to provide the best quality of the benchmark, including lesion segmentation masks and the corresponding types. This process contains two steps: screening and annotating. In the screening step, the radiologists first selected the patients of interest, as mentioned in the Participants and Screening Protocol Subsection. They then determined the type of lesions in each case study. In detail, HCC was determined using histopathology reports and evidence-based practice EASL/AASLD guidelines^33,34^. Non-HCC was based on histopathology reports or typical characteristics of imaging. The non-HCC group includes metastate, intrahepatic cholangiocarcinoma, hemangioma, cyst, abscess, focal nodular hyperplasia, adenoma, too small to characterize, undifferentiated, and other rare lesions.

In the annotating step, the radiologists then manually annotated the 3D region of the lesion using the free and open-source 3D Slicer imaging platform^35^. Based on the screening information, the radiologists used the CT phase specified in the screening step that provided the clearest depiction of the lesions to annotate all FLLs, except the one with a diameter smaller than 5 mm. Two radiologists independently annotated each lesion to guarantee the accurate mask of the FLLs. The annotation was accepted if two independent radiologists’ lesion masks reached a consensus with a Dice score greater than 0.8. Otherwise, the radiologists must discuss to confirm the annotated masks of lesions. Each lesion’s ground-truth mask was considered the intersection of the accepted annotations. Figure 1 visualizes the intersect annotated masks of the HCC and non-HCC lesions overlaid on the venous phase series. After the labeling process, we obtained 391 lesion masks from 253 patients, with 158 HCC tumors from 129 patients and 233 non-HCC lesions from 145 patients. The mean annotation consensus between radiologists in terms of Dice score was 0.92. A summary of the dataset is presented in Table 1. It is worth noting that rigid registration was performed to align the phases of each case study together. In particular, the arterial and delayed phases were automatically registered into the venous phase through our developed registration algorithm based on the iterative closest point technique.

**Table 1:** A summary of the dataset used in the experiments.

Types	No. patients	Gender dist. M|F	Mean age (range)	No. lesions	Annotation consensus
HCC	129	86|43	58.94 (23–86)	158	0.93 ± 0.06
Non-HCC	145	79|66	57.88 (18–86)	233	0.92 ± 0.06
Overall dataset	253	154|99	58.31 (18–86)	391	0.92 ± 0.06

Note that a patient can have both HCC and non-HCC lesions, leading to the summation of the number of patients with HCC and with non-HCC greater than the total number of patients. The annotation consensus between annotated was measured using Dice score.

We randomly separated the introduced dataset into the training set and test set with two criteria: (i) the ratio of the training set and test set was at most 8:2, and (ii) the distributions of lesion types and size between the sets were maximally equal. This results in the training set of 149 patients, with 99 HCC and 127 non-HCC lesions. The test set comprised 104 patients, with 59 HCC and 106 non-HCC lesions. Note that this study considered each lesion as a sample. Table 2 summarizes the sets.

**Table 2:** A summary of the training and test sets.

Types	No. patients	M|F	Age range	No. HCC	No. non-HCC
Train	149	93|56	(23–86)	99	127
Test	104	61|43	(18–86)	59	106
Total	253	154|99	(18–86)	158	233

## Methods

This was a retrospective study with the approval of the Human Research Ethics Committee at the University Medical Center of Ho Chi Minh City (number 93/GCN-HDDD, dated September 17, 2021). The written informed consent was waived by the Human Research Ethics Committee of the University Medical Center of Ho Chi Minh City. All methods were performed following the ethical standards of the Helsinki Declaration.

The rest of this section presents the workflow of the proposed radiomics feature-based model for classifying HCC and non-HCC lesions. We first describe the radiomics feature extraction and selection, and then classification algorithms.

### Radiomics feature extraction and selection

Radiomics features of the liver lesions can be extracted using the ROI on the segmented images. This study investigates features in original CT and wavelet domains for informative representations of liver diseases.

#### Feature extraction

Feature extraction has been performed in both original and wavelet domains. To mitigate the influence of inconsistent CT scan spacing within our dataset, prior to the feature extraction, we employed a cubic spline interpolation technique to recalibrate CT images to a uniform spacing of 1 mm × 1 mm × 1 mm. Each phase image of the original CT domain contributes 100 attributes, including 18 first-order statistics features, 14 shape features, and 68 texture features. We detail a list of all the used radiomics features in the supplementary document.

The first-order features characterize the spatial distribution of voxel intensities within the ROI. Such features represent commonly used metrics, including mean, variance, skewness, entropy, and uniformity. They are computed using direct image intensities or based on the histogram of the liver lesion ROI.

The shape features, on the other hand, are independent of the intensity distribution and give the visual representation of the FLLs. Typical shape attributes include diameter, area, and volume. Furthermore, *elongation* and *flatness* are also included as potential shape-related biomarkers. The texture features are based on second-order statistics and described via the density histogram and the spatial locations of image pixels. Three types of textures including gray level co-occurrence matrix (GLCM)^36^, gray level run length matrix (GLRLM)^37^, and gray level size zone matrix (GLSZM)^38^ are considered in this study.

In addition to the original-domain features, we extract several features from wavelet-derived images, namely higher-order features. They are extracted based on the first-order statistics and second-order textural features. These features are captured from the wavelet-domain images transformed by applying high (H) or low (L) filters in each of the three dimensions of the CT image. For the first level of wavelet decomposition, the filtering processing results in a total of 8 wavelet-filtered images: wavelet-LHL, wavelet-LHH, wavelet-HLL, wavelet-LLH, wavelet-HLH, wavelet-HHH, wavelet-HHL, and wavelet-LLL. Each wavelet-filtered image contributes 86 features (100 features excluding 14 shape features). Thus, one-level wavelet decomposition aggregates 688 radiomics features into the feature set. Higher-level wavelet decomposition further increases the number of radiomics features used for representing the hepatic lesions.

#### Feature selection

Feature selection plays a crucial role in the radiomics feature-based CAD models as this task produces compact but representative features that lead to improved interpretation, prediction, and generalization. In general, the feature selection can be performed using several techniques, including filter-based, wrapper-based, or model-based methods^39^. The filter-based methods select useful features by considering the statistical properties of the features. One widely-used filtering technique is the *feature variance thresholding*, which works by examining the feature variances and removes those with low values, i.e., likely containing little information. Another common technique, namely the *feature correlation thresholding* checks for features that have high correlations with others and eliminates one of them as they tend to contain redundant information.

The wrapper-based methods, on the other hand, employ an appointed model (regressor or classifier) to select features. The idea is to repeatedly train the selected model that contains its parameters, namely, *weights* or *coefficients*. At the first time, the model is trained using all the features. Then, the features are selected based on their important scores/ranks corresponding to the large absolute weights or coefficients. Note that the features selected by the wrapper-based methods may be sub-optimal due to being subjective to the nominated model.

In this study, we investigate the model-based techniques as they perform feature selection in the process of model construction. In particular, we consider feature selection as an ill-posed problem and introduce an efficient technique based on sparse representation to identify a compact but informative subset of features. It is worth noting here that the feature selection is regarded as an ill-posed or under-determined problem because the number of training samples (*M*) is far fewer than the number of features/variables (*N*)—$M \ll N$, especially for multiphase radiomics feature selection problems. This ill-posed problem can be addressed efficiently using the least squares (LS) optimization with regularizations.

Let us denote the supervised learning task having *M* training samples $\{ ({\textbf{x}}_i, y_i), i = 1, \dots , M\}$; each ${\textbf{x}}_i \in {\mathbb {R}}^N$ is an *N*-dimensional feature vector, and $y_i \in \{0, 1\}$ is a class label. It is worth noting here that for model performance improvement and outlier impact reduction, the feature vector ${\textbf{x}}_i$ is standardized to make sure it has a mean zero and a unit standard deviation,

1 $$\begin{aligned} {\textbf{x}}_i = \frac{{\textbf{x}}_i - \mu }{\sigma }, \end{aligned}$$

where $\mu$ and $\sigma$ are, respectively, the mean and standard deviation of the feature vector ${\textbf{x}}_i$.

Constructing the target vector ${\textbf{y}} = [ y_1, y_2, \dots , y_M ]^T \in {\mathbb {R}}^M$ and the feature matrix ${\textbf{X}} = [ {\textbf{x}}_1, {\textbf{x}}_2, \dots , {\textbf{x}}_M]^T \in {\mathbb {R}}^{M \times N}$. Defining the $\ell _2$-norm of a generic vector ${\textbf{x}} \in {\mathbb {R}}^N$ as $\Vert {\textbf{x}}\Vert _2 = (\sum ^N_{i = 1} x^2_i)^{1/2}$, the features can be selected by solving the following $\ell _2$-norm regularized LS problem:

2 $$\begin{aligned} \varvec{\theta } = \arg \min _{\varvec{\theta }} ( \Vert {\textbf{y}} - {\textbf{X}} \varvec{\theta } \Vert ^2_2 \, \, + \, \, \lambda \Vert \varvec{\theta }\Vert _2 ), \end{aligned}$$

where $\varvec{\theta } \in {\mathbb {R}}^{N}$ is the parameter vector (weights/coefficients) and $\lambda$ is a hyperparameter. Problem (2) is known as the *ridge regression* in the statistics literature. This problem consists of two terms. The first one is the LS term that attempts to fit the estimated response to the target, and the second term is the $\ell _2$ regularizer used to prevent the parameter values from increasing largely.

Using the $\ell _2$ regularizer can alleviate the *over-fitting* issue, but this model cannot yield a compact feature subset. The reason is the ridge regressor does not guarantee a sparse solution for the parameter vector $\varvec{\theta }$. To enforce model *sparsity*, we can replace $\ell _2$ with $\ell _1$ regularizer as

3 $$\begin{aligned} \varvec{\theta } = \arg \min _{\varvec{\theta }} ( \Vert {\textbf{y}} - {\textbf{X}} \varvec{\theta } \Vert ^2_2 \, \, + \, \, \lambda \Vert \varvec{\theta }\Vert _1 ). \end{aligned}$$

In (3), the $\ell _1$-norm of vector $\varvec{\theta }$ is defined as the sum of the absolute of its entries: $\Vert \varvec{\theta }\Vert _1 = \sum _{i = 1}^N | \theta _i |$ . This $\ell _1$-norm regularization promotes the sparsity by driving many entries of $\varvec{\theta }$ to be zeros. The non-zero entries ${\theta _i}$ in $\varvec{\theta }$ corresponds to the important features ${{\textbf{x}}_i}$ in ${\textbf{X}}$, which are considered to be the *relevant vector machine* (RVM)^40^. This sparsity-based model is similar to the LASSO technique in the statistics literature, and is robust to problems with the presence of many irrelevant features^41^. Note that the sparsity level, i.e., the number of nonzero values *K* ($K \ll N$) is governed by the hyperparameter $\lambda$. Increasing $\lambda$ leads to a sparser model and thus obtains fewer relevant features. In contrast, decreasing $\lambda$ means selecting more features. In practice, this important hyperparameter can be determined using searching techniques with cross-validation, such as grid-search, random-search^42^, or Bayesian optimization^43^.

The sparsity-based regression model in (3) is suitable for the problem where the target vector ${\textbf{y}}$ contains continuous entries. However, in our case, the vector ${\textbf{y}}$ comprises variables with only two states of 1 and 0 representing the HCC and non-HCC classes, respectively. Thus, extending the model in (3) is crucial to make it more efficient for our problem. The extension can be made by employing the statistical logistic regression method to model the probability of the target output. At the same time, we aim to keep the $\ell _1$ regularizer to maintain the model sparsity and RVM property. Integrating the logistic model and sparse representation may enhance the feature selection performance.

The proposed logistic sparsity-based regression can be modeled using the Bayesian framework. In particular, the probability distribution of the target $y_i$ given the feature vector ${\textbf{x}}_i$ can be expressed as

4 $$\begin{aligned} p(y_i = 1 | {\textbf{x}}_i, \varvec{\theta }) = \sigma (\varvec{\theta }^T {\textbf{x}}_i) = \frac{1}{1 + \exp (-\varvec{\theta }^T {\textbf{x}}_i)}. \end{aligned}$$

Here, $\sigma (\cdot )$ is the *logistic sigmoid* function. The prior distribution is introduced on the parameter $\varvec{\theta }$ using the Laplacian function given by

5 $$\begin{aligned} p(\varvec{\theta }) = (\lambda /2)^N \exp (-\lambda \Vert \varvec{\theta } \Vert _1). \end{aligned}$$

Using the likelihood function in (4) and prior distribution in (5), we can estimate the parameters $\varvec{\theta }$ by the *maximum a posteriori* (MAP). Note that maximizing the posterior function is equivalent to minimizing the negative of the log of this function, and thus we have the following optimization model:

6 $$\begin{aligned} \min _{\varvec{\theta }} \left\{ f(\varvec{\theta }) = \sum _{i = 1}^M - \log p(y_i| {\textbf{x}}_i, \varvec{\theta }) + \lambda \Vert \varvec{\theta } \Vert _1 \right\} . \end{aligned}$$

Problem (6) can be solved efficiently via proximal splitting methods^44^. By splitting, the first term in (6) is convex and differentiable and the second term is $\ell _1$-regularization which has a closed-form solution using *soft-thresholding* or *shrinkage* technique^45–47^. We detail the algorithm to solve Problem (6) in the supplementary document.

It is worth noting that the logistic sparsity regression (LSR) and LASSO are used for feature selection, but LSR is more suitable for HCC and non-HCC classification. LASSO primarily focuses on feature selection, whereas LSR excels in both feature selection and classification tasks due to its logistic regression foundation. Furthermore, LASSO’s linearity in (3) does not consider the non-linearities in the relationship between the wavelet-radiomics features and target classes. In contrast, LSR’s logistic formulation in (6) accommodates these complexities, enabling it to model intricate radiomics-target relationships.

### Classification of HCC and non-HCC

To differentiate between HCC and non-HCC lesions, we can apply any binary classification technique to the selected radiomics features. In this study, we aim to investigate the effects of using radiomics features extracted from the wavelet domain and their combination with those extracted from the original CT image. Furthermore, we evaluate the effectiveness of the proposed logistic sparsity-based model for both feature selection and classification. Therefore, we consider different popular classifiers, including the proposed LSR, SVM, and MLP.

The proposed LSR model in (6) can be considered as an extension of the widely used LR classifier in machine learning (ML). Note that for liver disease prediction, the standard LR was one of the prominent techniques employed in several studies, including^11,14,48,49^. This study uses the LSR model for the radiomics feature selection and HCC and non-HCC classification.

The SVM classifier is a popular technique for solving classification, regression, and novelty detection problems. In the classification case, the SVM is a decision machine designed to map the training examples to the points in the feature space to maximize the margin between the categories. The key feature of the SVM is that its object function not only maximizes the margin between the two classes but also minimizes a measure of the error on the training set^50,51^. In liver lesion classification, the state-of-the-art results obtained by SVM have been reported in numerous studies, including^11,52,53^.

The MLP is a fully connected feed-forward neural network used extensively in classification and regression. Compared to the LR and SVM, MLP is capable of yielding more complex decision boundaries. A comprehensive introduction to the MLP is given in^54^. For liver lesion classification, MLP has been used in several works, including^31,55,56^.

## Experimental results and discussion

This section presents the experimental results, performance analysis and comparison for the different important aspects of the wavelet-radiomics features-based approach to classifying HCC and non-HCC, including feature selection, the effect of combining different domain features, comparison of different feature selection models, analysis of using different wavelet families for optimal filter identification, and comparison with several deep CNN-based models. First, we give the experimental setup , then describe the results, analysis, and discussions on the study findings.

### Experimental protocol

Radiomics features were extracted in both original CT and wavelet-filtered images. For the original CT domain, feature extraction were performed for all the three phases, which results in 300 features. For the wavelet-domain feature extraction, the Haar filter was employed with one-level wavelet decomposition, leading to a set of 2064 texture features. All the radiomics features were obtained using pyradiomics, a python package for extracting radiomics features from medical imaging^57^.

To evaluate the performances of the different models, standard performance metrics for binary classification problems were used, including $\text{F}_1$ score and the area under the curve (AUC) of receiver operating characteristic (ROC). The $\text{F}_1$ score is the harmonic mean of the precision and recall. The AUC is a performance measure that provides the capability of distinguishing between the classes at different threshold levels^58^.

To assess the statistical significance of the proposed method, this study utilized the independent two-sample t-test^59^ and DeLong’s test on the AUC measures. These statistical tests determine whether the disparity between the means is likely to have occurred by chance (null hypothesis) or is statistically significant (alternative hypothesis), based on the calculated *p*-value. A *p*-value (*p*) less than 0.05 signifies rejection of the null hypothesis at a 95% confidence level. In the context of our experiments, this implies a significant distinction in the AUC measures between our proposed methods and the compared methods.

### Relevant feature selection: dominance of wavelet-derived features over original-domain features

This experiment aims to examine the proposed LSR model for feature selection and the contribution of different domain features to the selected subset features. In doing so, a total of *N* = 2364 features extracted from both the original and wavelet domains was used as input for feature selection. Since the feature selection was performed on the training set, these features represent the key characteristics for $M = 226$ training samples. The feature matrix ${\textbf{X}}$ is therefore of size $M \times N = 226~\times$ 2364, and the target vector ${\textbf{y}}$ is of size $M \times 1 = 226 \times 1$.

To perform feature selection using the LSR in (6), it is vital to tune a suitable hyperparameter $\lambda$. This hyperparameter can be determined using grid or random search techniques, which are known to be computationally expensive. To overcome this limitation, we used a 10-time repeated fivefold cross-validation (CV) with Bayesian optimization (BO). BO is capable of providing a principled technique to direct the search for a global optimization function (maximizing AUC metric in our case). By building a probabilistic model for the objective function, the search is effective with an acquisition function to choose candidate samples for the next objective function evaluation. It has been shown in^42,43^ that BO obtained better results in far fewer evaluations than its grid-search and random-search counterparts.

**Figure 2:**
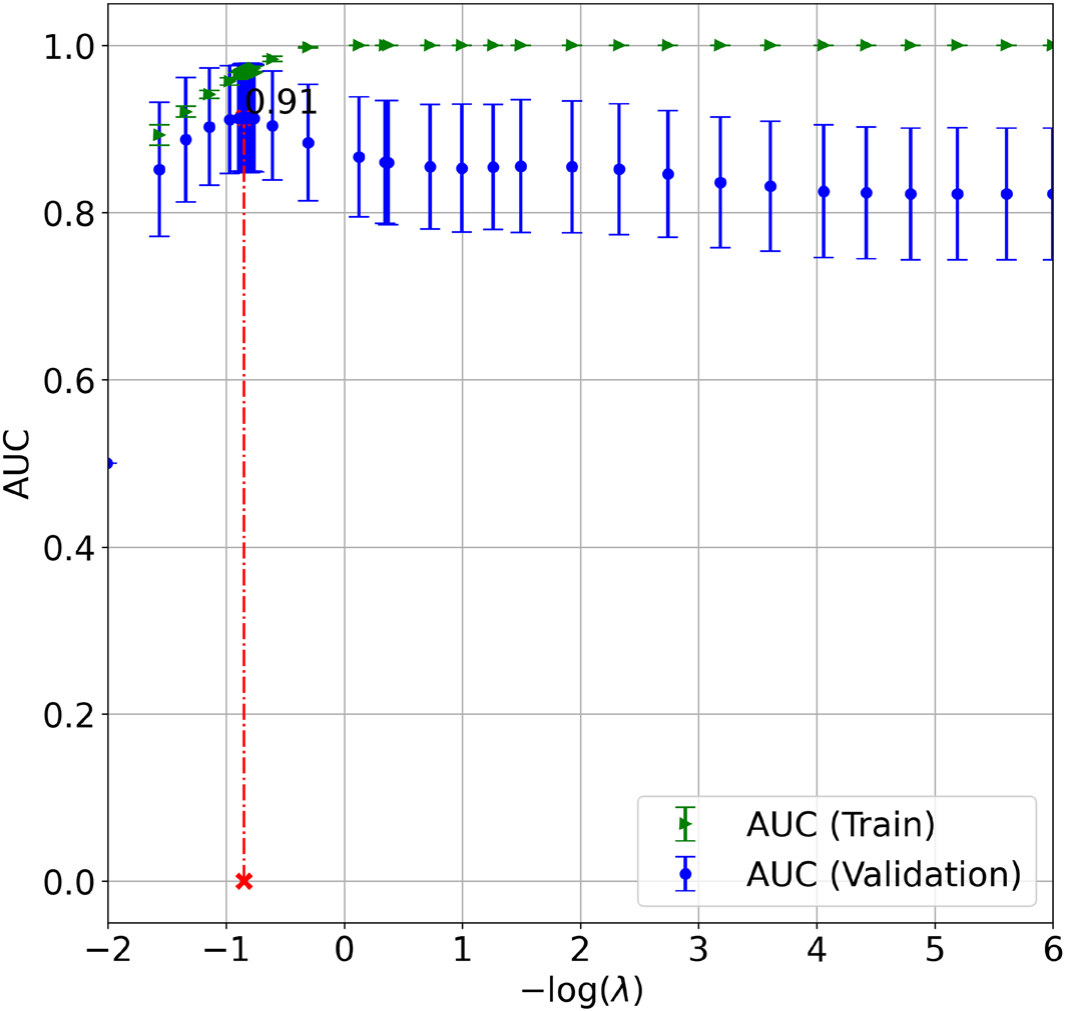
Bayesian optimization with a 10-time repeated fivefold cross-validation searching for the hyperparameter $\lambda$ used in the proposed logistic sparsity-based model based on maximum AUC criteria; the optimal regularization strength hyperparameter $\log (\lambda ) = 0.84$ was chosen at the maximum value of AUC = 0.91.

Figure 2 shows the performance metric AUC as a function of the hyperparameter $\lambda$. Here, the boundary search for this hyperparameter was initialized as $[10^{-6}~\mathrm {-}~10^{6}]$. It can be observed that the BO technique was very effective in that it can lead the searching direction to the potential space containing the optimum value. Once the search was finalized, the optimal hyperparameter $\log (\lambda ) = 0.84$ was found at the maximum value of AUC = 0.91.

Using the obtained hyperparameter, the proposed LSR model selected the features by solving Problem (6). After convergence, the parameter vector $\varvec{\theta }$ has only $K = 29$ nonzero coefficients out of the total of *N* = 2,364 entries ($K / N = 1.23\%$). This means that the proposed LSR model selected only 29 features from a total of 2364 features extracted from both original and wavelet domains. Table 3 lists such the selected features corresponding to the non-zero coefficients. It can be observed that the features extracted from both the original and wavelet domains were selected. However, the texture features from the wavelet domain tend to dominate those from the original counterpart. The wavelet domain contributes 20 out of 29 features ($68.97\%$), while the original domain makes up 9 out of 29 features ($31.03\%$). This implies that the wavelet-domain features play a very crucial role in the HCC and non-HCC classification performance, especially when combined with those extracted from the original domain.

**Table 3:** The most relevant radiomics features selected by the proposed logistic sparsity-based regression model.

Phases	Feature domains	Feature types	Feature names	Ranks	Coefficients
Venous	Original	GLSZM	GrayLevelNonUniformityNormalized	1	0.611
Arterial	Original	GLSZM	LowGrayLevelZoneEmphasis	2	− 0.530
Arterial	Wavelet-LLH	GLSZM	GrayLevelNonUniformityNormalized	3	0.362
Delayed	Original	GLSZM	GrayLevelNonUniformityNormalized	4	0.338
Venous	Wavelet-LHL	GLSZM	GrayLevelNonUniformity	5	0.315
Arterial	Wavelet-LHL	GLDM	DependenceVariance	6	− 0.285
Delayed	Original	GLDM	DependenceNonUniformityNormalized	7	0.209
Delayed	Original	GLCM	InverseVariance	8	-0.207
Venous	Wavelet-HLL	GLSZM	SizeZoneNonUniformityNormalized	9	− 0.181
Venous	Wavelet-LHH	GLSZM	SmallAreaHighGrayLevelEmphasis	10	0.170
Delayed	Original	GLDM	DependenceEntropy	11	− 0.159
Venous	Original	GLSZM	LowGrayLevelZoneEmphasis	12	− 0.143
Delayed	Wavelet-LHL	GLRLM	LongRunLowGrayLevelEmphasis	13	− 0.127
Delayed	Original	GLSZM	SmallAreaLowGrayLevelEmphasis	14	− 0.121
Venous	Wavelet-LLL	GLDM	DependenceNonUniformityNormalized	15	− 0.120
Delayed	Wavelet-LHL	GLDM	LargeDependenceLowGrayLevelEmphasis	16	− 0.110
Venous	Wavelet-LLH	GLSZM	SmallAreaEmphasis	17	0.109
Arterial	Wavelet-HLH	GLSZM	SmallAreaLowGrayLevelEmphasis	18	0.102
Venous	Original	GLRLM	LongRunLowGrayLevelEmphasis	19	0.087
Delayed	Wavelet-HLL	GLSZM	LowGrayLevelZoneEmphasis	20	− 0.085
Venous	Wavelet-LHL	GLRLM	ShortRunLowGrayLevelEmphasis	21	− 0.084
Delayed	Wavelet-HLH	FIRST-ORDER	Maximum	22	0.032
Venous	Wavelet-HLL	GLSZM	ZoneEntropy	23	0.029
Delayed	Wavelet-HLH	GLCM	JointEnergy	24	− 0.027
Arterial	Wavelet-HHH	GLRLM	LongRunLowGrayLevelEmphasis	25	0.019
Delayed	Wavelet-LLL	GLCM	Imc1	26	0.016
Venous	Wavelet-LLL	GLDM	DependenceEntropy	27	0.010
Venous	Wavelet-LLH	GLSZM	SmallAreaLowGrayLevelEmphasis	28	0.005
Venous	Wavelet-LHL	GLSZM	ZoneEntropy	29	0.001

These features correspond to the estimated dominant non-zero coefficients.

Furthermore, we find that all the three phases contribute to the selected feature subset. This 29-feature selected subset comprises 13 features from venous phase, 11 features from delayed phase, and 5 features from arterial phase. This result indicates that all the phases contain essential features necessary for screening HCC, and multiphase processing tends to be needed for performing HCC diagnosis.

### Analysis and comparison of different models for radiomics feature selection

In this experiment, we examine the performances of the different feature selection techniques for HCC and non-HCC discrimination with three different classifiers, i.e. logistic sparsity regression, multi-layer perceptron, and support vector machine. Here, we considered four major approaches: filter-based, wrapper-based, dimensional reduction with PCA, and model-based techniques. For filter-based methods, we implemented two popular techniques, namely the feature variance thresholding (FVT) and the feature correlation thresholding (FCT), that have been employed in^20^ for classifying HCC and hepatic hemangioma. Note that the FVT requires a pre-defined threshold $\tau _\text{v}$ for pruning the features with variances smaller than $\tau _\text{v}$. Similarly, the FCT needs a pre-defined threshold $\tau _\text{c}$ to identify the level of high-correlation between two features. In our experiment, we set $\tau _\text{v} = 0.5$ and $\tau _\text{c} = 0.99$. The wrapper-based approach, on the other hand, sticks to an appointed model to rank the features. Here we tested the wrapper method using the LR and random forest (RF) as these models are efficient for important feature rankings.

For the model-based techniques, together with the proposed logistic sparsity regression model, we also evaluated its variants of *logistic ridge regression* and *logistic elastic-net regression*. While the logistic sparsity regression uses $\ell _1$-regularizer and the logistic ridge regression employs $\ell _2$-regularizer, the logistic elastic-net regression enforces both the $\ell _1$ and $\ell _2$ penalties on the model parameters. For comparison, we tested here the LASSO model employing $\ell _1$ for sparse feature selection used for HCC identification^20^. Furthermore, we tested the widely-used PCA method, which reduces the number of features and retains the variance in the features. In our experiment, the PCA reduces the features but retains 99% variances.

Table 4 lists the results and performance metrics obtained by a 10-time repeated fivefold cross-validation for the different classifiers using the features selected by the different feature selection techniques. Here, all the feature selection techniques were performed on the training subset with the full 2,364 features extracted from both the original and wavelet domains. It can be observed that among the tested feature selection methods, the proposed logistic sparsity regression was the most efficient model. This model selected only 29 relevant features out of the total 2,364 features (1.23% of the full features), and yielded the highest $\text{F}_1$ and AUC metrics. The proposed logistic sparsity regression followed by the logistic sparsity classifier was found to obtain the highest $\text{F}_1$ score of 0.89 (95% CI 0.87–0.90), and AUC = 0.96 (95% CI 0.95–0.96).

**Table 4:** Performance metrics in terms of $\text{F}_1$ and AUC by the different classifiers using radiomics features selected by the different selection methods.

Classifiers	Feature selection models	No. selected/total features	$\varvec{\text{F}_1}$ (95% CI)	AUC (95% CI)
LSR	Proposed logistic sparsity	**29**/2364 (1.23%)	**0.89** (0.87–0.90)	**0.96** (0.95–0.96)
	Logistic ridge$^{*,+}$	796/2364 (33.67%)	0.84 (0.83–0.85)	0.92 (0.91–0.93)
	Logistic elastic-net$^{*,+}$	290/2364 (12.27%)	0.81 (0.80–0.83)	0.90 (0.89–0.91)
	LASSO^20^$$^{+}$$	30/2364 (1.27%)	0.89 (0.87–0.90)	0.95 (0.94–0.96)
	PCA^30^$$^{*,+}$$	194/2364 (8.21%)	0.66 (0.64–0.68)	0.75 (0.73–0.77)
	FVT & FCT^20^$$^{*,+}$$	887/2364 (37.52%)	0.73 (0.71–0.75)	0.82 (0.81–0.84)
	Wrapper LR^48^$$^{*,+}$$	30/2364 (1.27%)	0.78 (0.76–0.79)	0.82 (0.81–0.84)
	Wrapper RF^60^$$^{*,+}$$	30/2364 (1.27%)	0.82 (0.81–0.84)	0.88 (0.87–0.89)
MLP	Proposed logistic sparsity	29/2364 (1.23%)	**0.87** ( 0.86–0.89)	**0.94** ( 0.93–0.95)
	Logistic ridge$^{*,+}$	796/2364 (33.67%)	0.82 ( 0.80–0.83)	0.90 ( 0.89–0.92)
	Logistic elastic-net$^{*,+}$	290/2364 (12.27%)	0.82 ( 0.81–0.84)	0.91 ( 0.89–0.92)
	LASSO^20^	30/2364 (1.27%)	0.87 ( 0.86–0.88)	0.94 ( 0.93–0.95)
	PCA^30^$$^{*,+}$$	194/2364 (8.21%)	0.64 ( 0.62–0.66)	0.70 ( 0.68–0.72)
	FVT & FCT^20^$$^{*,+}$$	887/2364 (37.52%)	0.77 ( 0.75–0.79)	0.84 ( 0.83–0.86)
	Wrapper LR^48^$$^{*,+}$$	30/2364 (1.27%)	0.75 ( 0.74–0.77)	0.83 ( 0.82–0.85)
	Wrapper RF^60^$$^{*,+}$$	30/2364 (1.27%)	0.82 ( 0.81–0.84)	0.89 ( 0.88–0.91)
SVM	Proposed logistic sparsity	29/2364 (1.23%)	**0.89** ( 0.88–0.90)	**0.96** ( 0.95–0.97)
	Logistic ridge$^{*,+}$	796/2364 (33.67%)	0.83 ( 0.81–0.84)	0.91 ( 0.90–0.92)
	Logistic elastic-net$^{*,+}$	290/2364 (12.27%)	0.77 ( 0.75–0.79)	0.86 ( 0.85–0.88)
	LASSO^20^$$^{+}$$	30/2364 (1.27%)	0.88 ( 0.87–0.89)	0.95 ( 0.94–0.96)
	PCA^30^$$^{*,+}$$	194/2364 (8.21%)	0.65 ( 0.63–0.67)	0.73 ( 0.71–0.75)
	FVT & FCT^20^$$^{*,+}$$	887/2364 (37.52%)	0.71 ( 0.69–0.73)	0.79 ( 0.77–0.81)
	Wrapper LR^48^$$^{*,+}$$	30/2364 (1.27%)	0.77 ( 0.76–0.79)	0.82 ( 0.80–0.83)
	Wrapper RF^60^$$^{*,+}$$	30/2364 (1.27%)	0.82 ( 0.81–0.84)	0.88 ( 0.86–0.89)

The methods marked with asterisk ($*$) and/or plus (+) symbols indicate statistical significance compared to the proposed LSR method using the same classifier at a confidence level of 95%, as determined by the t-test and/or DeLong’s test, respectively.

The significant values, compared to the corresponding group in the first column, are in bolds.

On the other hand, logistic ridge or elastic-net produced lower performance metrics though they selected many more features. The AUCs produced by the logistic ridge and elastic-net are 0.92 (95% CI 0.91–0.93), and AUC = 0.90 (95% CI 0.89–0.91), respectively. Compared with these techniques, the LSR model enhances the performance with statistical significance ($p < 0.0001$). The LASSO produced good performances as it is a sparse feature selector. It yielded an AUC of 0.95 (95% CI 0.94–0.96). The proposed LSR enhances the AUC mean in comparison with LASSO, but not statistical significance ($p = 0.14$) by the t-test. In contrast, PCA was found to be ineffective for extracting informative features for HCC and non-HCC classification. The wrapper-based methods with LR and RF using the 30 most important features obtained reasonable performance metrics.

**Figure 3:**
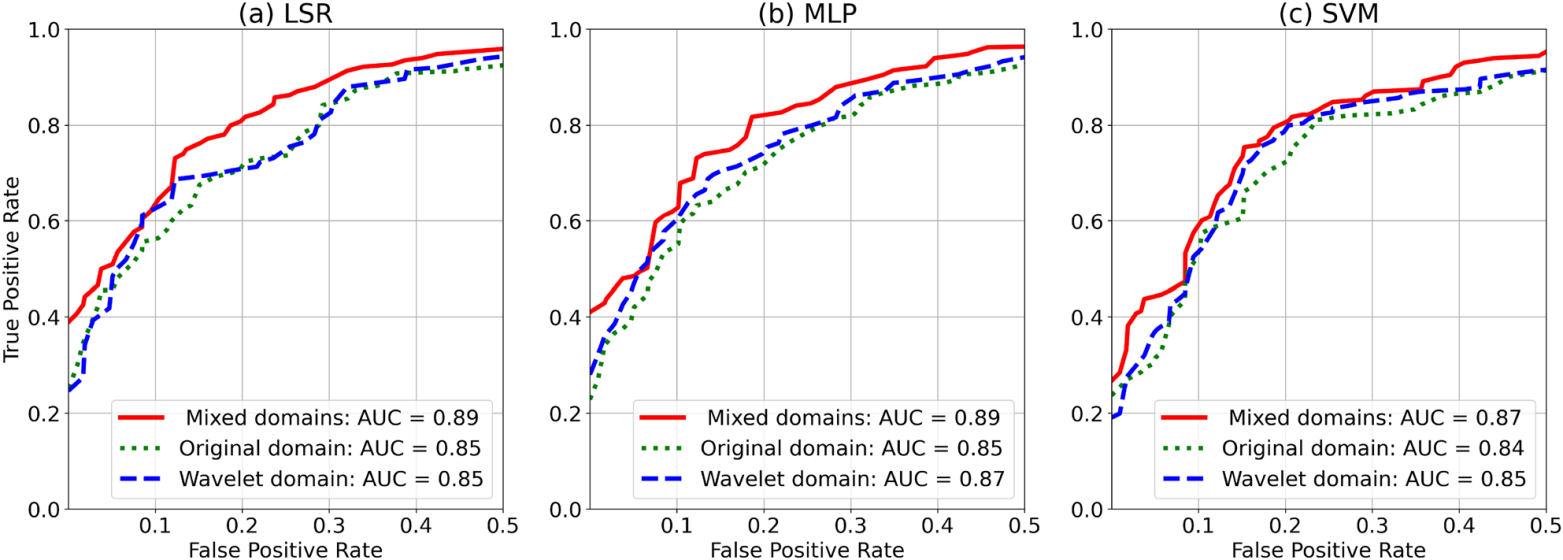
AUC ROC produced by the different classifiers using radiomics features extracted and selected from the mixed, original, and wavelet domains. Here the reverse biorthogonal 6.8 wavelet filter was used for the extraction of the radiomics features.

### Effects of wavelet-filtered features on classification performance

In this experiment, we aim to analyze the effects of using features extracted from different domains and their combinations on the performances of classifying HCC and non-HCC. To this end, we compare the prediction capabilities using three radiomics feature sets: (1) those extracted from both the original and wavelet domains, (2) those extracted from the original domain only, and (3) those extracted from the wavelet domain only. For each feature set, the important features are first selected by the best feature selection performer of the logistic sparsity regression, followed by the prediction using the different classifiers.

Table 5 depicts the performance metrics obtained using the three domain feature sets by the different classifiers evaluated on the training and test sets. On the training set, the model was evaluated with a 10-time repeated tenfold cross-validation. On the test set, the prediction was performed with a 200-time sampling replacement bootstrapping technique. This technique randomly draws data points with replacement from the original test set to create multiple test sets of equal size. For each of these bootstrapped sets, we assessed the model’s AUC performance. This process yielded a distribution of AUC values, considering variations in the test set composition. From this distribution, we calculated performance metrics, including the mean AUC and a 95% CI.

**Table 5:** The performance metric AUC obtained on the training set and test set by the different classifiers using radiomics features extracted from the different image domains.

Classifiers	Domain features	No. selected/total features	AUC (95% CI)	
			Training set	Test set
LSR	Wavelet and original domains	29/2364 (1.23%)	**0.96** (0.95–0.96)	**0.87** (0.82–0.92)
	Original domain$^{*,+}$	26/300 (8.67%)	0.92 (0.91–0.93)	0.85 (0.80–0.91)
	Wavelet domain$^{+}$	36/2064 (1.74%)	0.90 (0.89–0.91)	0.86 (0.80–0.91)
MLP	Wavelet and original domains	29/2364 (1.23%)	**0.96** (0.95–0.96)	**0.87** (0.82–0.92)
	Original domain$^{*,+}$	26/300 (8.67%)	0.93 (0.92–0.94)	0.85 (0.79–0.90)
	Wavelet domain$^{+}$	36/2064 (1.74%)	0.93 (0.91–0.94)	0.86 (0.80–0.91)
SVM	Wavelet and original domains	29/2364 (1.23%)	**0.96** (0.95–0.97)	**0.86** (0.79–0.92)
	Original domain$^{*,+}$	26/300 (8.67%)	0.93 (0.92–0.94)	0.83 (0.76–0.89)
	Wavelet domain$^{+}$	36/2064 (1.74%)	0.93 (0.92–0.94)	0.85 (0.79–0.90)

Here, the Haar wavelet filter was used for the extraction of the radiomics features. The domain features marked with asterisk ($*$) and/or plus (+) symbols indicate statistical significance compared to the mixed-domain features using the same classifier at a confidence level of 95%, as determined by the t-test and/or DeLong’s test, respectively.

The significant values, compared to the corresponding group in the first column, are in bolds.

The most noteworthy observation from the table is the improvement in the performance metrics obtained from the mixed-domain feature set, compared with those computed from the original-domain or wavelet-domain feature sets. For instance, using the LSR classifier, the mixture of wavelet and original feature set has an AUC of 0.96 (95% CI 0.95–0.96), whereas the original radiomics feature set yielded an AUC of 0.92 (95% CI 0.91–0.93), and the wavelet-domain features only obtained an AUC of 0.90 (95% CI 0.89–0.91). The improvement of the mixed-domain features over either the original domain or wavelet domain only is statistically significant ($p < 0.0001$), confirmed by both the t-test and DeLong’s test.

Similarly, on the test set, the prediction results show that combining the wavelet- and original-domain radiomics features enhances the classification performance compared with using the original-domain features only. This improvement is statistically significant ($p < 0.0001$), confirmed by DeLong’s test, though the 95% CIs overlap. However, the enhancement of the mixed-domain features over the wavelet-domain features is not statistically significant according to the t-test ($p > 0.05$ ).

**Figure 4:**
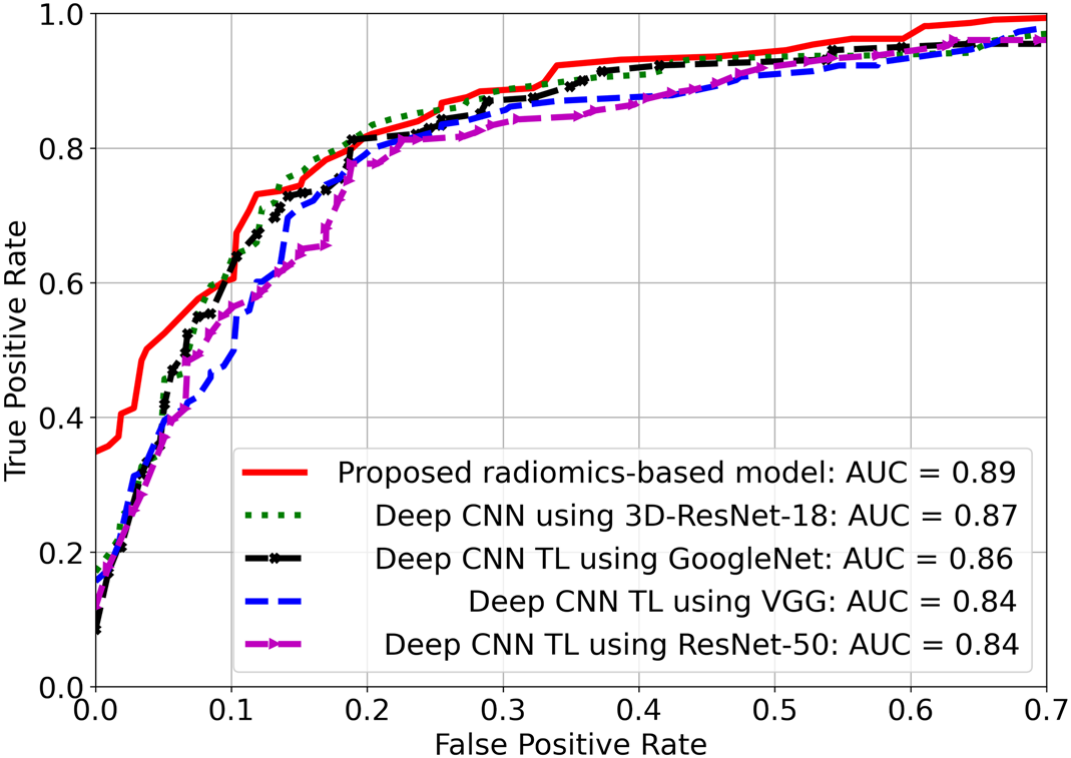
AUC ROC produced by the proposed radiomics features-based model and deep CNN-based approaches using different backbones for HCC and non-HCC classification.

### Effects of different wavelet-family radiomics features on classification performance

This section analyzes the effect of using different mother wavelet-based radiomics features on classifying HCC and non-HCC performance and aims to identify the most suitable wavelet family for this classification problem. We have followed^62^ to select four different wavelet families for radiomics feature extraction, including Haar, Daubechies 7, Biorthogonal 6.8, and Reverse biorthogonal 6.8. For each family, we performed one level wavelet decomposition and radiomics feature extraction, and then used LSR for feature selection and classification.

Table 6 lists the results in terms of the subset of features selected, and performance metric AUC on the training and test sets. It can be observed that the proposed wavelet-based radiomics feature model is capable of yielding satisfactory classification performances, regardless of the wavelet families used. In addition, combining the wavelet and original-domain features considerably enhances the AUC metrics. This observation is consistent among all the tested wavelet families. For these wavelet transforms, on the test set, the Reverse biorthogonal 6.8 is the most suitable filter and yields the highest AUC of 0.89 (95% CI 0.84–0.94), followed by the Haar wavelet with an AUC of 0.87 (95% CI 0.82–0.92). According to the t-test and DeLong’s test, the wavelet family of reverse biorthogonal 6.8 enhances the performance among the tested wavelet filters with statistical significance ($p < 0.0001$), even though their 95% CIs overlap.

**Table 6:** The performance metric AUC obtained by using the different wavelet family filters for radiomics feature extraction from the different image domains.

Wavelet filters	Domain features	No. selected/total features	AUC (95% CI)	
			Training set	Test set
Haar$^{*,+}$	Wavelet and original domains	29/2364 (1.23%)	**0.96** (0.95–0.96)	**0.87** (0.82–0.92)
	Original domain	26/300 (8.67%)	0.92 (0.91–0.93)	0.85 (0.80–0.91)
	Wavelet domain	36/2064 (1.74%)	0.90 (0.89–0.91)	0.86 (0.80–0.91)
Daubechies 7$^{*,+}$	Wavelet and original domains	15/2364 (0.63%)	**0.94** ( 0.93–0.95)	**0.86** (0.80–0.91)
	Original domain	26/300 (8.67%)	0.93 ( 0.92–0.94)	0.83 (0.76–0.89)
	Wavelet domain	39/2064 (1.89%)	0.92 ( 0.91–0.94)	0.80 (0.74–0.86)
Biorthogonal 6.8$^{*,+}$	Wavelet and original domains	27/2364 (1.14%)	**0.94** ( 0.93–0.95)	**0.87** (0.82–0.92)
	Original domain	29/300 (9.67%)	0.93 ( 0.92–0.94)	0.85 (0.80–0.90)
	Wavelet domain	36/2064 (1.74%)	0.92 (0.91–0.93)	0.86 (0.81–0.92)
Reverse bior. 6.8	Wavelet and original domains	32/2364 (1.35%)	**0.96** (0.95–0.96)	**0.89** (0.84–0.94)
	Original domain	30/300 (10.00%)	0.93 (0.92–0.94)	0.85 (0.79–0.90)
	Wavelet domain	64/2064 (3.10%)	0.93 (0.92–0.94)	0.86 (0.80–0.91)

The wavelet family marked with asterisk ($*$) and/or plus (+) symbols indicate statistical significance compared to Reverse biorthogonal 6.8 with the corresponding feature domain at a confidence level of 99.99% ($p < 0.0001$), as determined by the t-test and/or DeLong’s test, respectively.

The significant values, compared to the corresponding group in the first column, are in bolds.

For illustration, Fig. 3 shows the AUC of ROC obtained using the radiomics features extracted and selected from the different image domains followed by the different classifiers. It can be observed that employing the wavelet-domain radiomics features improves the AUC. Furthermore, combining the different radiomics feature domains leads to enhanced AUC compared to using either original or wavelet radiomics features only. This enhancement can be justified by the fact that the combination of the two domain features gives a more informative and discriminative representation of HCC and non-HCC lesions.

### Performance comparison with deep CNN-based models

This section presents the performance comparison between the proposed wavelet radiomics-based model and other existing deep CNN-based approaches for addressing the problem of HCC and non-HCC classification. As the deep CNN-based models tend to yield unsatisfactory results in the cases of limited training data samples, for fair comparison, we consider here the deep CNN models using transfer learning techniques. The transfer learning techniques rely on the CNN backbones pretrained on other tasks and inherit the trained weights, i.e., knowledge transfer for solving a new task. The transfer learning techniques using several CNN backbones, including VGG^6^, ResNet-50^7^, and GoogleNet^8^ are considered here. Furthermore, since recent 3D CNN models have shown to enhance the liver lesion classification performances, we implemented here the deep 3D CNN model using 3D ResNet-18 architecture^9^. The Python code using the pre-trained deep CNNs is given in the supplementary document.

Table 7 depicts the performance metrics in terms of the $\text{F}_1$ and AUC on the test set by the different models. It can be observed that the proposed wavelet-based radiomics feature model is capable of yielding satisfactory classification performances. It produced an $\text{F}_1$ score of 0.80 (95%CI 0.73–0.86). This score is comparable to those yielded by the deep CNN-TL using GoogleNet and deep CNN using 3D ResNet-18 methods. In terms of AUC, the proposed wavelet radiomics-based model considerably enhances the performance and yields the highest AUC of 0.89 (95% CI 0.83–0.93), followed by the deep CNN using 3D-ResNet-18 model with an AUC of 0.87 (95% CI 0.82–0.93), and deep CNN TL using GoogleNet with an AUC of 0.86 (95% CI 0.79–0.92). Figure 4 further illustrates the AUCs yielded by the different classification methods. Compared with the deep CNN-based models, the proposed wavelet radiomics-based approach yields higher AUC metrics with statistical significance ($p < 0.0001$), as confirmed by both the t-test and DeLong’s test.

**Table 7:** Performance metrics in terms of $\text{F}_1$ and AUC by the different approaches based on radiomics features and deep CNN features for HCC identification.

Classification models	$\varvec{\text{F}_1}$ (95% CI)	AUC (95% CI)
Proposed radiomics-based method	0.80 (0.73–0.86)	**0.89** (0.83–0.93)
Deep CNN TL using VGG^6^$$^{*,+}$$	0.78 (0.73–0.85)	0.84 (0.77–0.90)
Deep CNN TL using ResNet-50^7,61^$$^{*,+}$$	0.78 (0.71–0.84)	0.84 (0.76–0.90)
Deep CNN TL using GoogleNet^8^$$^{*,+}$$	0.80 (0.74–0.86)	0.86 (0.79–0.92)
Deep CNN using 3D-ResNet-18^9^$$^{*,+}$$	0.80 (0.74–0.87)	0.87 (0.82–0.93)

The methods marked with asterisk ($*$) and plus (+) symbols indicate statistical significance compared to the proposed method at a confidence level of 99.99%, as determined by the t-test and DeLong’s test, respectively.

The significant AUC value is in bold.

## Conclusion

This paper presented an analysis of using radiomics features extracted from multiphase CT images to address the problem of classifying HCC and non-HCC liver lesions. Through the experimental results, analysis and comparisons, the following significant findings can be drawn from this study. First, combining the wavelet-derived texture features with the original CT image features significantly improves the discriminative capability between the HCC and non-HCC lesions. Second, the proposed logistic sparsity regression with Bayesian optimization is capable of selecting compact and relevant radiomics features for HCC and non-HCC representations. The proposed logistic sparsity-based model is the most suitable feature selector among the tested feature selection counterparts and yields higher performance metrics in terms of AUC. Third, in the limited training data cases, the proposed wavelet radiomics-based features approach is comparable if not outperforms several recent deep CNN-based models used for HCC and non-HCC classification.

### Supplementary Information

Supplementary Information.

## Data Availability

The dataset generated and analyzed during this study is protected patient information. Some data may be available for research purposes from the corresponding author upon reasonable request.

## References

[1] Sung H (2021). Global cancer statistics 2020: GLOBOCAN estimates of incidence and mortality worldwide for 36 cancers in 185 countries. CA Cancer J. Clin..

[2] Schütte K, Schulz C, Malfertheiner P (2014). Hepatocellular carcinoma: Current concepts in diagnosis, staging and treatment. Gastrointest. Tumors.

[3] Navin PJ, Venkatesh SK (2019). Hepatocellular carcinoma: State of the art imaging and recent advances. J. Clin. Transl. Hepatol..

[4] Lysdahlgaard S (2022). Comparing radiomics features of tumour and healthy liver tissue in a limited CT dataset: A machine learning study. Radiography.

[5] Tian J, Dong D, Liu Z, Wei J (2021). Radiomics and Its Clinical Application.

[6] Meng D (2017). Liver fibrosis classification based on transfer learning and fcnet for ultrasound images. IEEE Access.

[7] Ben-Cohen A (2018). Fully convolutional network and sparsity-based dictionary learning for liver lesion detection in CT examinations. Neurocomputing.

[8] Balagourouchetty L, Pragatheeswaran JK, Pottakkat B, Ramkumar G (2020). GoogLeNet-based ensemble FCNet classifier for focal liver lesion diagnosis. IEEE J. Biomed. Health Inform..

[9] Zhou J (2021). Automatic detection and classification of focal liver lesions based on deep convolutional neural networks: A preliminary study. Front. Oncol..

[10] Nayantara PV, Kamath S, Manjunath K, Rajagopal K (2020). Computer-aided diagnosis of liver lesions using CT images: A systematic review. Comput. Biol. Med..

[11] Nayak A (2019). Computer-aided diagnosis of cirrhosis and hepatocellular carcinoma using multi-phase abdomen CT. Int. J. Comput. Assist. Radiol. Surg..

[12] Li S (2018). A pilot study using kernelled support tensor machine for distant failure prediction in lung SBRT. Med. Image Anal..

[13] Sreeja, P. & Hariharan, S. Image analysis for the detection and diagnosis of hepatocellular carcinoma from abdominal CT images. In *Proc. Intelligent Communication and Computational Technologies*, 107–117, 10.1007/978-981-10-5523-2_11 (2018).

[14] Chang C-C (2017). Computer-aided diagnosis of liver tumors on computed tomography images. Comput. Methods Programs Biomed..

[15] Wang S, Summers RM (2012). Machine learning and radiology. Med. Image Anal..

[16] Lambin P (2017). Radiomics: The bridge between medical imaging and personalized medicine. Nat. Rev. Clin. Oncol..

[17] Parmar C, Grossmann P, Bussink J, Lambin P, Aerts HJWL (2015). Machine learning methods for quantitative radiomic biomarkers. Sci. Rep..

[18] Lambin P (2012). Radiomics: Extracting more information from medical images using advanced feature analysis. Eur. J. Cancer.

[19] Lian C, Ruan S, Denœux T, Jardin F, Vera P (2016). Selecting radiomic features from FDG-PET images for cancer treatment outcome prediction. Med. Image Anal..

[20] Wu J (2019). Radiomics-based classification of hepatocellular carcinoma and hepatic haemangioma on precontrast magnetic resonance images. BMC Med. Imaging.

[21] Khan RA, Luo Y, Wu F-X (2022). Machine learning based liver disease diagnosis: A systematic review. Neurocomputing.

[22] Sayed GI, Hassanien AE, Schaefer G (2016). An automated computer-aided diagnosis system for abdominal CT liver images. Procedia Comput. Sci..

[23] Anter, A. M. & Hassenian, A. E. Normalized multiple features fusion based on PCA and multiple classifiers voting in CT liver tumor recognition. In *Proc. Advances in Soft Computing and Machine Learning in Image Processing*, 113–129, 10.1007/978-3-319-63754-9_6 (2018).

[24] Alahmer H, Ahmed A (2016). Computer-aided classification of liver lesions from CT images based on multiple ROI. Procedia Comput. Sci..

[25] Balagourouchetty L, Pragatheeswaran JK, Pottakkat B, Govindarajalou R (2018). Enhancement approach for liver lesion diagnosis using unenhanced CT images. IET Comput. Vis..

[26] Duda D, Kretowski M, Bezy-Wendling J (2013). Computer-aided diagnosis of liver tumors based on multi-image texture analysis of contrast-enhanced CT selection of the most appropriate texture features. Stud. Logic Gramm. Rhetoric.

[27] Sun J (2015). Automatic computer-aided diagnosis of liver disease based on multi-cascade and multi-featured classifier. J. Med. Imaging Health Inform..

[28] de Lima Thomaz R (2018). Novel Mahalanobis-based feature selection improves one-class classification of early hepatocellular carcinoma. Med. Biol. Eng. Comput..

[29] Jiang H, Zheng R, Yi D, Zhao D (2013). A novel multiinstance learning approach for liver cancer recognition on abdominal CT images based on CPSO-SVM and IO. Comput. Math. Methods Med..

[30] Yu, L., Wang, C., Cheng, S. & Guo, L. Establishment of computer-aided diagnosis system for liver tumor CT based on SVM. In *Proc. IEEE International Conference on Data Science in Cyberspace*, 710–715, 10.1109/DSC.2018.00113 (2018).

[31] Gunasundari S, Ananthi MS (2012). Comparison and evaluation of methods for liver tumor classification from CT datasets. Int. J. Comput. Appl..

[32] Chen E-L, Chung P-C, Chen C-L, Tsai H-M, Chang C-I (1998). An automatic diagnostic system for CT liver image classification. IEEE Trans. Biomed. Eng..

[33] American Association for the Study of Liver Diseases. Management of Hepatocellular Carcinoma. Tech. Rep. (2022). (Accessed 22 Aug 2022).

[34] Aubé C (2017). EASL and AASLD recommendations for the diagnosis of HCC to the test of daily practice. Liver Int..

[35] 3D Slicer image computing platform. https://slicer.org.

[36] Haralick RM, Shanmugam K, Dinstein I (1973). Textural features for image classification. IEEE Trans. Syst. Man. Cybern..

[37] Tang X (1998). Texture information in run-length matrices. IEEE Trans. Image Process..

[38] Thibault, G. *et al.* Texture indexes and gray level size zone matrix application to cell nuclei classification. In *Proc. International Conference on Pattern Recognition and Information Processing*, 1–6 (2009).

[39] Guyon I, Elisseeff A (2003). An introduction to variable and feature selection. J. Mach. Learn..

[40] Bishop CM (2006). Pattern Recognition and Machine Learning.

[41] Tibshirani R (1996). Regression shrinkage and selection via the lasso. J. Roy. Stat. Soc.: Ser. B (Methodol.).

[42] Bergstra, J., Bardenet, R., Bengio, Y. & Kégl, B. Algorithms for hyper-parameter optimization. In *Proc. Advances in Neural Information Processing Systems*, vol. 24, 2546–2554 (2011).

[43] Snoek, J., Larochelle, H. & Adams, R. P. Practical Bayesian optimization of machine learning algorithms. In *Proc. Advances in Neural Information Processing Systems*, vol. 25, 2951–2959 (2012).

[44] Combettes, P. L. & Pesquet, J.-C. Proximal splitting methods in signal processing. In *Fixed-Point Algorithms for Inverse Problems in Science and Engineering*, 185–212, 10.1007/978-1-4419-9569-8_10 (2011).

[45] Lee, S.-I., Lee, H., Abbeel, P. & Ng, A. Efficient L1 regularized logistic regression. vol. 21 (2006).

[46] Meier L, Geer SVD, Bühlmann P (2008). The group lasso for logistic regression. J. R. Stat. Soc. Ser. B Stat. Methodol..

[47] Beck A, Teboulle M (2009). A fast iterative shrinkage-thresholding algorithm for linear inverse problems. SIAM J. Imag. Sci..

[48] Jing R (2021). A wavelet features derived radiomics nomogram for prediction of malignant and benign early-stage lung nodules. Sci. Rep..

[49] Shan Q-Y (2016). Focal lesions in fatty liver: If quantitative analysis facilitates the differentiation of atypical benign from malignant lesions. Sci. Rep..

[50] Vapnik, V., Golowich, S. & Smola, A. Support vector method for function approximation, regression estimation and signal processing. In *Proc. Advances in Neural Information Processing Systems*, vol. 9, 281-287 (1996).

[51] Burges, C. J. C. & Schölkopf, B. Improving the accuracy and speed of support vector machines. In *Proc. Advances in Neural Information Processing Systems*, vol. 9, 375–381 (1996).

[52] Chen, X. *et al.* A dual-attention dilated residual network for liver lesion classification and localization on CT images. In *IEEE International Conference on Image Processing*, 235–239, 10.1109/ICIP.2019.8803009 (2019).

[53] Xu SS-D, Chang C-C, Su C-T, Phu PQ (2019). Classification of liver diseases based on ultrasound image texture features. Appl. Sci..

[54] Haykin S (2008). Neural Networks and Learning Machines.

[55] Hwang YN, Lee JH, Kim GY, Jiang YY, Kim SM (2015). Classification of focal liver lesions on ultrasound images by extracting hybrid textural features and using an artificial neural network. Bio-Med. Mater. Eng..

[56] Das A, Acharya UR, Panda SS, Sabut S (2019). Deep learning based liver cancer detection using watershed transform and Gaussian mixture model techniques. Cogn. Syst. Res..

[57] van Griethuysen JJ (2017). Computational radiomics system to decode the radiographic phenotype. Can. Res..

[58] Fawcett T (2006). An introduction to ROC analysis. Pattern Recogn. Lett..

[59] Dietterich TG (1998). Approximate statistical tests for comparing supervised classification learning algorithms. Neural Comput..

[60] Liu D (2021). Optimization and evaluation of the random forest model in the efficacy prediction of chemoradiotherapy for advanced cervical cancer based on radiomics signature from high-resolution T2 weighted images. Arch. Gynecol. Obstet..

[61] Marya NB (2021). Application of artificial intelligence using a novel EUS-based convolutional neural network model to identify and distinguish benign and malignant hepatic masses. Gastrointest. Endosc..

[62] Soufi M, Arimura H, Nagami N (2018). Identification of optimal mother wavelets in survival prediction of lung cancer patients using wavelet decomposition-based radiomic features. Med. Phys..

