# Supplementary material for: Wavelet radiomics features from multiphase CT images for screening hepatocellular carcinoma: analysis and comparison
Source: Sci Rep. 2023 Nov 10;13:19559. doi: 10.1038/s41598-023-46695-8 (PMC10638447; doi:10.1038/s41598-023-46695-8)
Supplement: Supplementary file 1 — Supplementary Information. [file 41598_2023_46695_MOESM1_ESM.pdf]

Wavelet radiomics features from multiphase CT images for screening hepatocellular carcinoma: analysis and comparison

Van Ha Tang[1,2], Soan T. M. Duong[1,2,*], Chanh D. Tr. Nguyen[1,3], Thanh M. Huynh[1,3], Vo T. Duc[4], Chien Phan[4], Huyen Le[4], Trung Bui[5], and Steven Q. H. Truong[1,3]

[1]VinBrain JSC, Vietnam
[2]Le Quy Don Technical University, Vietnam
[3]VinUniversity, Vietnam
[4]Ho Chi Minh City Medicine and Pharmacy University, Vietnam
[5]Adobe Research, USA
[*]Corresponding author, email: soanduong@lqdtu.edu.vn

Supplementary information

In this supplementary document, we present three additional pieces of information: a list of used radiomics features, logistic sparsity algorithm, and Python code for training and validating the deep convolutional neural networks (DCNNs) used in the paper.

List of used radiomics features

Feature extraction has been performed in both original CT and wavelet-filtered images. Each image of the original CT domain contributes 100 attributes, including 18 first-order statistics features, 14 shape features, and 68 texture features. However, each wavelet-filtered image contributes only 86 features (100 features excluding 14 shape features). The list of all the radiomics features that have been used is listed in the below tables.

Supplementary Table 1: The 18 radiomics first-order statistics features.

Feature types (abbre.)	Feature names
FIRST-ORDER	10Percentile
FIRST-ORDER	90Percentile
FIRST-ORDER	Energy
FIRST-ORDER	Entropy
FIRST-ORDER	InterquartileRange
FIRST-ORDER	Kurtosis
FIRST-ORDER	Maximum
FIRST-ORDER	MeanAbsoluteDeviation
FIRST-ORDER	Mean
FIRST-ORDER	Median
FIRST-ORDER	Minimum
FIRST-ORDER	Range
FIRST-ORDER	RobustMeanAbsoluteDeviation
FIRST-ORDER	RootMeanSquared
FIRST-ORDER	Skewness
FIRST-ORDER	TotalEnergy
FIRST-ORDER	Uniformity
FIRST-ORDER	Variance

Supplementary Table 2: The 14 radiomics shape features.

Feature types (abbre.)	Feature names
SHAPE	Elongation
SHAPE	Flatness
SHAPE	LeastAxisLength
SHAPE	MajorAxisLength
SHAPE	Maximum2DDiameterColumn
SHAPE	Maximum2DDiameterRow
SHAPE	Maximum2DDiameterSlice
SHAPE	Maximum3DDiameter
SHAPE	MeshVolume
SHAPE	MinorAxisLength
SHAPE	Sphericity
SHAPE	SurfaceArea
SHAPE	SurfaceVolumeRatio
SHAPE	VoxelVolume

Supplementary Table 3: The 22 radiomics gray level co-occurrence matrix (GLCM) features.

Feature types (abbre.)	Feature names
GLCM	Autocorrelation
GLCM	JointAverage
GLCM	ClusterProminence
GLCM	ClusterShade
GLCM	ClusterTendency
GLCM	Contrast
GLCM	Correlation
GLCM	DifferenceAverage
GLCM	DifferenceEntropy
GLCM	DifferenceVariance
GLCM	JointEnergy
GLCM	JointEntropy
GLCM	Imc1
GLCM	Imc2
GLCM	Idm
GLCM	Idmn
GLCM	Id
GLCM	Idn
GLCM	InverseVariance
GLCM	MaximumProbability
GLCM	SumEntropy
GLCM	SumSquares

Supplementary Table 4: The 16 radiomics gray level run length matrix (GLRLM) features.

Feature types (abbre.)	Feature names
GLRLM	GrayLevelNonUniformity
GLRLM	GrayLevelNonUniformityNormalized
GLRLM	GrayLevelVariance
GLRLM	HighGrayLevelRunEmphasis
GLRLM	LongRunEmphasis
GLRLM	LongRunHighGrayLevelEmphasis
GLRLM	LongRunLowGrayLevelEmphasis
GLRLM	LowGrayLevelRunEmphasis
GLRLM	RunEntropy
GLRLM	RunLengthNonUniformity
GLRLM	RunLengthNonUniformityNormalized
GLRLM	RunPercentage
GLRLM	RunVariance
GLRLM	ShortRunEmphasis
GLRLM	ShortRunHighGrayLevelEmphasis
GLRLM	ShortRunLowGrayLevelEmphasis

Supplementary Table 5: The 16 radiomics gray level size zone matrix (GLSZM) features.

Feature types (abbre.)	Feature names
GLSZM	GrayLevelNonUniformity
GLSZM	GrayLevelNonUniformityNormalized
GLSZM	GrayLevelVariance
GLSZM	HighGrayLevelZoneEmphasis
GLSZM	LargeAreaEmphasis
GLSZM	LargeAreaHighGrayLevelEmphasis
GLSZM	LargeAreaLowGrayLevelEmphasis
GLSZM	LowGrayLevelZoneEmphasis
GLSZM	SizeZoneNonUniformity
GLSZM	SizeZoneNonUniformityNormalized
GLSZM	SmallAreaEmphasis
GLSZM	SmallAreaHighGrayLevelEmphasis
GLSZM	SmallAreaLowGrayLevelEmphasis
GLSZM	ZoneEntropy
GLSZM	ZonePercentage
GLSZM	ZoneVariance

Supplementary Table 6: The 14 radiomics gray level dependence matrix (GLDM) features.

Feature types (abbre.)	Feature names
GLDM	DependenceEntropy
GLDM	DependenceNonUniformity
GLDM	DependenceNonUniformityNormalized
GLDM	DependenceVariance
GLDM	GrayLevelNonUniformity
GLDM	GrayLevelVariance
GLDM	HighGrayLevelEmphasis
GLDM	LargeDependenceEmphasis
GLDM	LargeDependenceHighGrayLevelEmphasis
GLDM	LargeDependenceLowGrayLevelEmphasis
GLDM	LowGrayLevelEmphasis
GLDM	SmallDependenceEmphasis
GLDM	SmallDependenceHighGrayLevelEmphasis
GLDM	SmallDependenceLowGrayLevelEmphasis

Logistic sparsity algorithm

This section presents the computational steps for solving the logistic sparsity model in (1):

$$\min_{\theta} \left\{ f(\theta) = \sum_{i=1}^{M} -\log p(y_i|\mathbf{x}_i, \theta) + \lambda \|\theta\|_1 \right\}. \quad (1)$$

Before solving this ℓ_1 regularized optimization problem, let us consider the unregularized version, i.e., the objective function $f(\theta)$ with the first term only:

$$\min_{\theta} \sum_{i=1}^{M} -\log p(y_i|\mathbf{x}_i, \theta). \quad (2)$$

We now derive a technique based on Newton's method to solve Problem (2) and then extend the derivation to the sparsity regularized version.

Newton's method solves Problem (2) in an iterative manner. At each iteration, this method uses the current point for approximating the objective function with the second-order Taylor expansion and searching for a step direction. In particular, let θ_k be the estimate at the kth iteration. Newton's technique computes a step direction γ_k by the following evaluation at the current point θ_k,

$$\gamma_k = \theta_k - \mathbf{H}^{-1}(\theta_k)\,\mathbf{g}(\theta_k). \quad (3)$$

In (3), $\mathbf{H}(\theta_k)$ and $\mathbf{g}(\theta_k)$ are, respectively, the Hessian and gradient of the objective function evaluated at the current estimate θ_k. Once the step direction is obtained, the next estimate is computed as

$$\theta_{k+1} = (1-t)\,\theta_k + t\,\gamma_k. \quad (4)$$

Here, the step size $t \in [0,1]$ can be determined using a backtracking line search[1], that is designed to minimize the objective function given in (2).

As the aim is to solve the ℓ_1-regularized optimization problem (1) efficiently, we now show that instead of computing the step direction γ_k directly using (3), this point can be found as a solution to a weighted LS problem, see[2,3]. In doing so, let us define two auxiliary variables—a diagonal matrix $\mathbf{A}$ and a vector $\mathbf{z}$,

$$\begin{aligned} A_{i,i} &= \sigma(\theta_k^T\,\mathbf{x}_i)\,[1-\sigma(\theta_k^T\,\mathbf{x}_i)], \\ z_i &= \mathbf{x}_i^T\,\theta_k + \frac{[1-\sigma(y_i\,\theta_k^T\,\mathbf{x}_i)]\,y_i}{A_{i,i}}, \end{aligned} \quad (5)$$

for $i = 1,\ 2, \ldots, M$. Now, the Hessian and gradient are computed by $\mathbf{H}^{-1}(\theta_k) = -\mathbf{X}^T\,\mathbf{A}\,\mathbf{X}$, and $\mathbf{g}(\theta_k) = \mathbf{X}^T\,\mathbf{A}\,(\mathbf{z} - \mathbf{X}\,\theta_k)$. Equation (3) can therefore be rewritten as

$$\gamma_k = (\mathbf{X}^T\,\mathbf{A}\,\mathbf{X})^{-1}\,\mathbf{X}^T\,\mathbf{A}\,\mathbf{z}. \tag{6}$$

The result in (6) means that the step direction γ_k is the solution to the following weighted LS problem:

$$\gamma_k = \arg\min_{\gamma} \|(\mathbf{A}^{\frac{1}{2}}\,\mathbf{X})\,\gamma - \mathbf{A}^{\frac{1}{2}}\,\mathbf{z}\|_2^2. \tag{7}$$

For the ℓ_1 regularized optimization problem, we can augment this regularizer to obtain:

$$\gamma_k = \arg\min_{\gamma} \|(\mathbf{A}^{\frac{1}{2}}\,\mathbf{X})\,\gamma - \mathbf{A}^{\frac{1}{2}}\,\mathbf{z}\|_2^2 + \lambda\,\|\gamma\|_1. \tag{8}$$

Thus, our task now is to solve Problem (8) to obtain the step direction, and then do the line search to determine the step size and finally update the next estimate using (4). Problem (8) can be solved efficiently using the proximal splitting method and the soft-thresholding technique. Let $\mathbf{a}_k$ be an auxiliary variable computed at the kth iteration,

$$\mathbf{a}_k = \gamma_k - \alpha\,(\mathbf{A}^{\frac{1}{2}}\,\mathbf{X})^T\,[(\mathbf{A}^{\frac{1}{2}}\,\mathbf{X})\,\gamma - \mathbf{A}^{\frac{1}{2}}\,\mathbf{z}], \tag{9}$$

where the step size α is selected as $\alpha = 1/\|(\mathbf{A}^{\frac{1}{2}}\,\mathbf{X})\|_2^2$ for fast convergence—$\|(\mathbf{A}^{\frac{1}{2}}\,\mathbf{X})\|_2^2$ being the Lipschitz constant of the gradient of the first term in (8). Then, the solution to (8) is obtained by applying a soft-thresholding operator to the variable $\mathbf{a}_k$ with a threshold $\tau = \alpha\,\lambda$:

$$\gamma_{k+1} = \mathscr{T}(\mathbf{a}_k, \tau). \tag{10}$$

Here, $\mathscr{T}(\cdot)$ is the component-wise shrinkage function given by,

$$\mathscr{T}(x,\tau) = \mathrm{sgn}(x)\max(|x| - \tau, 0) = \frac{x}{|x|}\max(|x| - \tau, 0). \tag{11}$$

The steps of the proposed algorithm are summarized in Algorithm 1. Given the training set $\{\mathbf{x}_i, y_i\}$, for $i = 1,\ 2, \ldots, M$, the algorithm starts by initializing the parameter θ in Step 3, then estimates the step direction in Steps 6–10, follows by the update of the parameter in Step 12. As the algorithm minimizes the cost function $f(\theta)$, its convergence can be ascertained when the changes of this function are very small, i.e., smaller than a pre-defined $\mathrm{tol} = 10^{-4}$.

Algorithm 1: LSR for radiomics feature selection and HCC and non-HCC classification.

1: **Input:** M training samples $\{(\mathbf{x}_i, y_i), i = 1, \ldots, M\}$, the feature matrix $\mathbf{X} = [\mathbf{x}_1, \mathbf{x}_2, \ldots, \mathbf{x}_M]^T \in \mathbb{R}^{M\times N}$, hyperparameter λ, and a tolerance $\mathrm{tol} = 10^{-4}$, and MaxIter = 100.
2: **Output:** the parameter vector θ.
3: Initialize the parameter $\theta_0 = 0$, and iteration index $k = 0$.
4: **repeat**
5: Compute $\mathbf{A}$ and $\mathbf{z}$ using (5):
6: $A_{i,i} = \sigma(\theta_k^T\,\mathbf{x}_i)\,[1 - \sigma(\theta_k^T\,\mathbf{x}_i)]$,
7: $z_i = \mathbf{x}_i^T\,\theta_k + \frac{[1-\sigma(y_i\,\theta_k^T\,\mathbf{x}_i)]\,y_i}{A_{i,i}}$.
8: Estimate the step direction γ_{k+1} using (9) and (10):
9: $\mathbf{a}_k = \gamma_k - \alpha\,(\mathbf{A}^{\frac{1}{2}}\,\mathbf{X})^T\,[(\mathbf{A}^{\frac{1}{2}}\,\mathbf{X})\,\gamma - \mathbf{A}^{\frac{1}{2}}\,\mathbf{z}]$,
10: $\gamma_{k+1} = \mathscr{T}(\mathbf{a}_k, \tau)$.
11: Find the step size t by a backtracking line-search and update the parameter θ using (4):
12: $\theta_{k+1} = (1-t)\,\theta_k + t\,\gamma_{k+1}$.
13: Increment $k = k+1$, evaluate the objective function $f(\theta)$ in (1), and check for the stopping criterion.
14: **until** $(|f(\theta)^{k+1} - f(\theta)^k|/|f(\theta)^k| < \mathrm{tol})$ or $(k > \mathrm{MaxIter})$

Python code for training and inference of deep convolutional neural networks (DCNNs)

This section presents the main Python code used to train DCNNs using transfer learning. In particular, the DCNNs are used as fixed feature extractors. In other words, their weights are frozen except for the final fully connected layer. This last layer is replaced with our binary HCC and non-HCC classifier with random weights, and only this layer is trained. Here, we use the *PyTorch* deep learning library[4] to implement the classification task. First, we prepare the dataset by defining a class named *CustomImageDataset*. This class is inherited from the parent class of torch.utils.data.Dataset responsible for accessing and processing single instances of the data. Another concept is DataLoader, which is used to get data instances from the dataset, collect them in batches, and return them for usage in the training loop. The implementation of the CustomImageDataset is given below.

```python
import torch
import torch.nn as nn
from torch.utils.data import Dataset
from torchvision import datasets, models, transforms
from scipy import ndimage
import copy

# This class is designed for data augmentation (performing image rotation)
class Rotate_transform(object):
    # This method is to perform rotation on a single-phase image
    def rotate_data(self, volume):
            volume = volume.squeeze()
            volume = volume.permute(1, 2, 0)
            angles = [-20, -10, -5, 5, 10, 20]
            idx = torch.randint(0, len(angles), (1,))  # pick angles to rotate image randomly
            angle = angles[idx]
            max_vol = torch.max(volume)
            min_vol = torch.min(volume)
            volume = ndimage.rotate(volume, angle, reshape=False)
            volume = torch.from_numpy(volume)
            volume[volume < min_vol] = min_vol
            volume[volume > max_vol] = max_vol
            volume = volume.permute(2, 0, 1)
            return volume

    # This method is to apply the roration for all the three phase images
    # The call method enables instances to behave like functions
    def __call__(self, sample):
        img_venous, img_delay, img_arterial, label = sample
        img_venous   = self.rotate_data(img_venous)
        img_delay    = self.rotate_data(img_delay)
        img_arterial = self.rotate_data(img_arterial)
        sample =  img_venous, img_delay, img_arterial, label
        return sample

# A user-defined custom dataset class specifically designed for handling the multiphase CT image data.
# It is created to handle our own dataset class by subclassing torch.utils.data.Dataset.
class CustomImageDataset(Dataset):
    # Define a constructor: initialize the attributes:
    def __init__(self, imgs, labels, phase_lst, transform = None, target_transform = None):
        self.labels = labels
        self.imgs = imgs
        self.phase_lst = phase_lst
        self.transform = transform
        self.target_transform = target_transform

    # Customize the behavior of the len() function when applied to instances of the class
    def __len__(self):
        return len(self.labels)

    # allow instances of the class to be indexed and accessed, enabling object instances to support indexing and iteration.
    def __getitem__(self, idx):
        img_dict = dict()
        for phase_ in self.phase_lst:
            img = self.imgs[phase_][idx]
            label = self.labels[idx]
            image = normalize(img)
```

```
            label = torch.tensor(label)
            image = torch.from_numpy(image)
            image = image.permute(2, 0, 1)
            img_dict[phase_] = image
        sample = img_dict['venous'], img_dict['delay'], img_dict['arterial'], label
        if self.transform is not None:
            sample = self.transform(sample)
        return sample
```

Supplementary Listing 1. Define a CustomImageDataset for training and inference of the deep CNN models.

We now write a general function to train and validate a model, including scheduling the learning rate, tracking the training history, and saving the best model.

```
def train_validate_model(model, dataloaders, criterion, optimizer, scheduler,  num_epochs,
    checkpoint_file):
    val_acc_history = []
    train_acc_history = []
    val_loss_history = []
    train_loss_history = []
    training_history = {}

    best_model_wts = copy.deepcopy(model.state_dict())
    best_acc = 0.0
    best_loss = sys.float_info.max

    for epoch in range(num_epochs):
        print('Epoch {}/{}'.format(epoch, num_epochs - 1))
        print('-' * 10)
        # Each epoch has a training and validation phase
        for phase in ['train', 'val']:
            if phase == 'train':
                model.train()  # Set model to training mode
            else:
                model.eval()   # Set model to evaluate mode

            running_loss = 0.0
            running_corrects = 0

            # Iterate over data
            for inputs_img_v, inputs_img_d, inputs_img_a, labels in dataloaders[phase]:
                inputs_img_v = inputs_img_v.to(device)
                inputs_img_d = inputs_img_d.to(device)
                inputs_img_a = inputs_img_a.to(device)
                labels = labels.to(device)

                # zero the parameter gradients
                optimizer.zero_grad()

                # forward
                # track history if only in train
                with torch.set_grad_enabled(phase == 'train'):
                    # Get model outputs and calculate loss

                    outputs = model(inputs_img_v, inputs_img_d, inputs_img_a)
                    labels = labels.long()
                    loss = criterion(outputs, labels)
                    _, preds = torch.max(outputs, 1)

                    # backward and optimize only if in training phase
                    if phase == 'train':
                        loss.backward()
                        optimizer.step()

                # statistics
                running_loss += loss.item() * inputs_img_v.size(0)
                running_corrects += torch.sum(preds == labels.data)

            epoch_loss = running_loss / len(dataloaders[phase].dataset)
            epoch_acc = running_corrects.double() / len(dataloaders[phase].dataset)
```

```
            if epoch >= 1:
                print('{} Loss: {:.4f} Acc: {:.4f} Best Loss: {:.4f}'
                .format(phase, epoch_loss, epoch_acc, best_loss))
            else:
                print('{} Loss: {:.4f} Acc: {:.4f}'.format(phase, epoch_loss, epoch_acc))

            # deep copy the best model on the validation set
            if phase == 'val' and epoch_acc > best_acc:
                best_acc = epoch_acc
                best_model_wts = copy.deepcopy(model.state_dict())

            if phase == 'val' and epoch_loss < best_loss:
                best_loss = epoch_loss
                best_model_loss = copy.deepcopy(model.state_dict())

            # track the training history
            if phase == 'val':
                val_acc_history.append(epoch_acc)
                val_loss_history.append(epoch_loss)
            else:
                train_acc_history.append(epoch_acc)
                train_loss_history.append(epoch_loss)

        scheduler.step()
        print(f'learning rate: {scheduler.get_last_lr()[0]:.7f}')

    print('Training complete:')
    print('Best val Acc: {:4f}'.format(best_acc))
    print('Best val loss: {:4f}'.format(best_loss))

    # load best model weights
    model.load_state_dict(best_model_wts)


    # save best model weights with respect to loss:
    checkpoint = {'epoch': num_epochs,
                  'model_state_dict': best_model_loss,
                  'optimizer_state_dict': optimizer.state_dict(),
                  'loss': train_loss_history,
                  'val_loss': val_loss_history}

    torch.save(checkpoint, checkpoint_file)
    training_history = {'val_acc': val_acc_history,
                        'val_loss': val_loss_history,
                        'train_acc': train_acc_history,
                        'train_loss': train_loss_history}
    return model, training_history
```

Supplementary Listing 2. The training and validating procedure for the deep CNN models.

We now define a deep CNN model for the multiphase CT liver lesion imaging analysis for the binary classification of HCC and non-HCC. The model loads a pre-trained DCNN model including VGGNet[5], ResNet[6], DenseNet[7], and GoogleNet[8] and reset the final fully connected layer. Here, we customize it for processing our multiphase CT images.

```
# Define the class used to replace the last fully connected layer in pretrained models
class Identity(nn.Module):
    def __init__(self):
        super().__init__()

    def forward(self, x):
        return x

# Define a pretrained model for single-phase CT HCC and non-HCC classification
class Singlephase_PreTrained_Model(nn.Module):
    def __init__(self, model_name = None, num_classes = 2):
        super().__init__()
        if model_name == 'densenet':
            self.pretrained = models.densenet121(pretrained=True)
            for param in self.pretrained.parameters():  # freeze all the learned weights.
                param.requires_grad = False
```

```python
            self.num_ftrs = self.pretrained.classifier.in_features
            # set the last layer to the identity so the net becomes a feature extractor.
            self.pretrained.classifier = Identity()

        if model_name == 'resnet':
            self.pretrained = models.resnet50(pretrained=True)
            for param in self.pretrained.parameters():
                param.requires_grad = False
            self.num_ftrs = self.pretrained.fc.in_features
            self.pretrained.fc = Identity()

        if model_name == 'googlenet':
            self.pretrained = models.googlenet(pretrained = True)
            for param in self.pretrained.parameters():
                param.requires_grad = False
            self.num_ftrs = self.pretrained.fc.in_features
            self.pretrained.fc = Identity()

        if model_name == 'vgg':
            self.pretrained = models.vgg11_bn(pretrained = True)
            for param in self.pretrained.parameters():
                param.requires_grad = False
            self.num_ftrs = self.pretrained.classifier[6].in_features
            self.pretrained.classifier[6] = Identity()

        self.num_classes = num_classes
        self.relu = nn.ReLU()
        self.fc1 = nn.Linear(self.num_ftrs, 512)
        self.fc2 = nn.Linear(512, self.num_classes)
        self.Dropout = nn.Dropout(0.5)


    def forward(self, x):
        x = self.pretrained(x)
        x = x.view(-1,self.num_ftrs)
        x = self.relu(self.fc1(x))
        x = self.Dropout(x)
        x = self.fc2(x)
        return x
# Define a pretrained model for multiphase CT HCC and non-HCC classification
class Multiphase_PreTrained_Model(nn.Module):
    def __init__(self, model_name = None, num_classes = 2):
        super().__init__()
        self.pretrained_v = Singlephase_PreTrained_Model(model_name, num_classes)
        self.pretrained_d = Singlephase_PreTrained_Model(model_name, num_classes)
        self.pretrained_a = Singlephase_PreTrained_Model(model_name, num_classes)

    def forward(self, x_v, x_d, x_a):
        x_v = self.pretrained_v(x_v)
        x_d = self.pretrained_d(x_d)
        x_a = self.pretrained_a(x_a)
        x = (x_v + x_d + x_a) / 3
        return x
```

Supplementary Listing 3. Define a deep CNN model for the multiphase CT liver lesion HCC and non-HCC classification.

We can now use the given classes to make the Datasets and DataLoaders, perform necessary settings, and select the optimizer and criterion (loss function) to train and validate the model. The code is given below.

```python
# Settings for training the model:
num_classes = 2       # Number of classes in the dataset
batch_size = 32       # Batch size for training (can be changed depending on how much memory we have)
num_epochs = 500      # Number of epochs
learning_rate = 1e-3  # Learning rate
model_name = 'resnet' # Name of the pretrained model

# Define datasets and dataloaders:
phase_lst = ['venous', 'delay', 'arterial']
training_data = CustomImageDataset(imgs_valid_train, y_train, phase_lst, transform = transforms.Compose
    ([Rotate_transform()]))
```

```
test_data = CustomImageDataset(imgs_valid_test, y_test, phase_lst)
training_loader = torch.utils.data.DataLoader(training_data, batch_size = batch_size, shuffle = True)
test_loader = torch.utils.data.DataLoader(test_data, batch_size = batch_size, shuffle = False)
dataloaders_dict = {'train': training_loader, 'val': test_loader}

# Load the model, select optimizer, loss function, train and validate the model:
model = Multiphase_PreTrained_Model(model_name, num_classes)
device = torch.device("cuda:0" if torch.cuda.is_available() else "cpu")
model = model.to(device)
params_to_update = model.parameters()
optimizer = torch.optim.Adam(params_to_update, lr = learning_rate)
scheduler = torch.optim.lr_scheduler.StepLR(optimizer, step_size = 50, gamma = 0.1)
criterion = nn.CrossEntropyLoss()
checkpoint_file = model_name + "_checkpoint.pth"
model_best, training_history = train_validate_model(model,
                                                    dataloaders_dict,
                                                    criterion,
                                                    optimizer,
                                                    scheduler,
                                                    num_epochs = num_epochs,
                                                    checkpoint_file = checkpoint_file)
```

Supplementary Listing 4. Script for preparing the dataloaders and selecting the optimizer and loss function to train and validate the model.

References

1. Boyd, S. & Vandenberghe, L. *Convex optimization* (Cambridge University Press, 2004).

2. Minka, T. A comparison of numerical optimizers for logistic regression (2003).

3. Lee, S.-I., Lee, H., Abbeel, P. & Ng, A. Efficient L1 regularized logistic regression. vol. 21 (2006).

4. Paszke, A. *et al.* Pytorch: An imperative style, high-performance deep learning library. In *Advances in Neural Information Processing Systems*, 8024–8035 (Curran Associates, Inc., 2019).

5. Simonyan, K. & Zisserman, A. Very deep convolutional networks for large-scale image recognition. In *International Conference on Learning Representations* (2015).

6. He, K., Zhang, X., Ren, S. & Sun, J. Deep residual learning for image recognition. In *IEEE Conference on Computer Vision and Pattern Recognition (CVPR)*, 770–778, DOI: 10.1109/CVPR.2016.90 (2016).

7. Huang, G., Liu, Z., Van Der Maaten, L. & Weinberger, K. Q. Densely connected convolutional networks. In *IEEE Conference on Computer Vision and Pattern Recognition (CVPR)*, 2261–2269, DOI: 10.1109/CVPR.2017.243 (2017).

8. Szegedy, C. *et al.* Going deeper with convolutions. In *IEEE Conference on Computer Vision and Pattern Recognition (CVPR)*, 1–9, DOI: 10.1109/CVPR.2015.7298594 (2015).
